# Novel Method of Increased Efficiency Corn Drying on a Fixed Bed by Condensation

**DOI:** 10.3390/foods12051027

**Published:** 2023-02-28

**Authors:** Daping Fu, Wenfu Wu, Guiying Wang, Hong Xu, Feng Han, Zhe Liu

**Affiliations:** 1 College of Biological and Agricultural Engineering, Jilin University, 5988 Renmin Road, Changchun 130025, China; 2 College of Engineering and Technology, Jilin Agricultural University, 2888 Xincheng Street, Changchun 130118, China; 3 Jilin Business and Technology College, 1666 Cullen Lake Road, Changchun 130507, China; 4 College of Materials Science and Engineering, Jilin University, 5988 Renmin Road, Changchun 130025, China

**Keywords:** increased efficiency by condensation, energy-saving drying, energy efficiency, exergy efficiency, improvement potential rate, sustainability index

## Abstract

Exhaust air recycling is a simple and commonly used technique to save energy when using a dryer. The fixed-bed drying test device with increased efficiency by condensation is a clean and energy-saving drying test device developed by combining exhaust air recycling and condensation dehumidification technology. In this paper, through comparisons with or without exhaust air circulation using the single factor test of drying process parameters and the response surface test of corn drying on this test device to investigate the energy-saving effect and drying characteristics resulting from the novel drying method of increased efficiency by condensation. We drew the following main conclusions: (1) increased efficiency drying by condensation resulted in an energy savings of 32–56% compared with the conventional open hot air drying; and (2) during the increased efficiency corn drying by condensation, the mean energy and exergy efficiencies were within 31.65–51.26% and 41.69–63.52%, respectively, when the air temperature was in the 30–55 °C range, and they were 24.96–65.28% and 30.40–84.90%, respectively, when the air passed through the grain layer at 0.2–0.6 m/s; both of these increased with increasing air temperature, and decreased with increasing air velocity. These conclusions may constitute an important reference for investigating the energy-saving drying process of increased efficiency by condensation and developing relevant energy-saving drying equipment.

## 1. Introduction

Corn is a staple food crop in China, with this country ranking second globally in corn production and consumption [1]. Drying the harvested corn in a timely manner significantly contributes to the process of corn storage and processing. Since drying is energy-intensive and highly polluting, clean and energy-saving drying to extend the shelf life of corn is an important research objective. Corn is primarily dried via continuous hot air drying, and a massive amount of heat is liberated by exhaust air emissions. Since coal-fired furnaces are the main drying heat sources, an enormous amount of polluting gases and fumes are emitted during this process [2]. Therefore, recovering the exhaust air and employing a clean drying heat source can help conserve energy and reduce the emissions in the drying process.

Exhaust air recovery or recycling is one of the main approaches to conserve energy and reduce emissions when operating dryers [3,4,5]. A study suggested that when the winter temperature was –10 °C, the temperature required for recovering the exhaust gas emitted by a continuous flow grain dryer during corn drying was in the 34–40 °C range, representing a temperature rise of 44–50 °C, which corresponds to 500,000 kcal/h of heat or 100 kg/h of coal. By substituting the open hot air drying with a fully enclosed structure-based exhaust air recovery process, the recovery of vegetable matter and dust can reach 90%, and the energy consumption can be reduced by more than 3%. A pilot-scale wood drying test conducted using a convection dryer showed that exhaust air recycling could markedly improve the exergy efficiency of the dryer by more than two times. This indicates that exhaust air recovery in dryers can noticeably promote energy conservation and emission reduction. Furthermore, the exergy efficiency of a dryer with an exhaust air circulation system increases as the air circulation ratio increases [6].

As China remains committed to pursuing clean, low-carbon, and green development and reaching the goals of peaking carbon dioxide emissions before 2030 and achieving carbon neutrality before 2060, energy efficient and eco-friendly farm machines will be vigorously developed. In a dryer, the dryer heat source is one of the main factors affecting energy conservation and emission reduction. Due to the rising demand for a clean dryer heat source, oil-, gas-, and biomass-fired hot-blast stoves and heat pumps have emerged. Studies in China and abroad have indicated that biomass-fired hot-blast stoves and heat pumps are the preferred heat sources for reducing pollution and the drying costs [7,8,9,10,11,12]. The alcohol-based mixed fuel hot-blast stove [13] and the electrothermal energy storage hot-blast stove [14,15] are emerging as clean, low-carbon, energy-saving, and eco-friendly heat sources for grain dryers in China; however, they are still in the market development stage. Electricity, which can be generated from a clean energy source, possesses the inherent advantage of a low initial investment when employed as the heat source for grain dryers [16], however, it cannot be widely used due to its large power configuration.

Increased efficiency drying by condensation, a new type of hot air drying, is a process in which the exhaust air is recovered, condensed, dehumidified, and recycled. More specifically, the drying medium flows in a closed loop in the drying system. This method possesses the prominent advantages of cleanliness, energy conservation, and intelligent control during the drying process [17,18,19]. Its novelty lies in the utilization of both the residual heat of exhaust air and the latent heat of condensation through moderate condensation of recycled exhaust air and exergic analysis of the drying process directing the selection of drying process parameters, with the purpose of clean and energy-saving drying. Fixed-bed drying is traditionally employed to dry corn [20]. In recent years, there were many studies on drying process simulations of corn fixed-bed drying based on the MSU (Michigan State University) model [21,22] and partial differential equations drying model [23], and some effective drying simulation research methods were established. For the purpose of energy saving drying, based on the increased efficiency by condensation hot air drying combined with fixed-bed drying, a fixed-bed drying test device of increased efficiency by condensation was designed to investigate and optimise the fixed-bed drying process of increased efficiency by condensation. Here, we compared conventional open hot air drying and increased efficiency drying by condensation using corn drying test under the same drying conditions, using the response surface method test and the corn drying test of increased efficiency by condensation under different drying conditions to verify the energy-saving advantage of the novel drying method, and the influence of drying process parameters on the drying characteristics and exergy characteristics of the corn drying process.

## 2. Materials and Methods

### 2.1. Materials and Test and Analytical Methods

The corn used for the test was Ketai 881 corn freshly harvested from Quanyan Town, Erdao District, Changchun, Jilin, China. The corn was harvested and threshed in the field, washed to remove the impurities, and placed in sealed bags, followed by freezing in a freezer and stored at −15 °C. During the test, an appropriate amount of the sample was removed and placed in an environment at 23 °C for approximately 1 h to remove the frost on the surface. Then, an additional 500 g of sample was weighed with a high precision electronic scale of the Yingheng brand to conduct the drying test. The sample weight before and after the test was measured by the electronic scale and moisture was measured using a PM-8188 grain moisture meter produced by KETT Japan. In the drying process, the total weight method was used to detect corn moisture in real time [24]. The test data of the comparative test with or without exhaust air circulation and the single factor test were statistically analysed using Excel 2013, and the response surface method test data were statistically analysed using Design-Expert v8.0.6.1.

The comparative test with or without exhaust air circulation was performed with an air velocity across the grain layer of 0.4 m/s, condensation ratio of 0.8, and hot air temperature of 30, 35, 40, 45, 50, and 55 °C. The single factor experiment was performed at six temperatures (30, 35, 40, 45, 50, and 55 °C), five air velocities across the grain layer levels (0.2, 0.3, 0.4, 0.5, and 0.6 m/s), and five condensation ratios (0.4, 0.6, 0.8, 1.0, and 1.2). The single factor air temperature test data were the same as the comparative test data with or without exhaust air circulation. In the single factor air velocity test, the air temperature was 40 °C and condensation ratio was 0.8. In the single factor condensation ratio test, the air temperature was 40 °C and the air velocity across the grain layer was 0.3 m/s. A rotary combination design was used for response surface test.

### 2.2. Test Device

**Figure 1:**
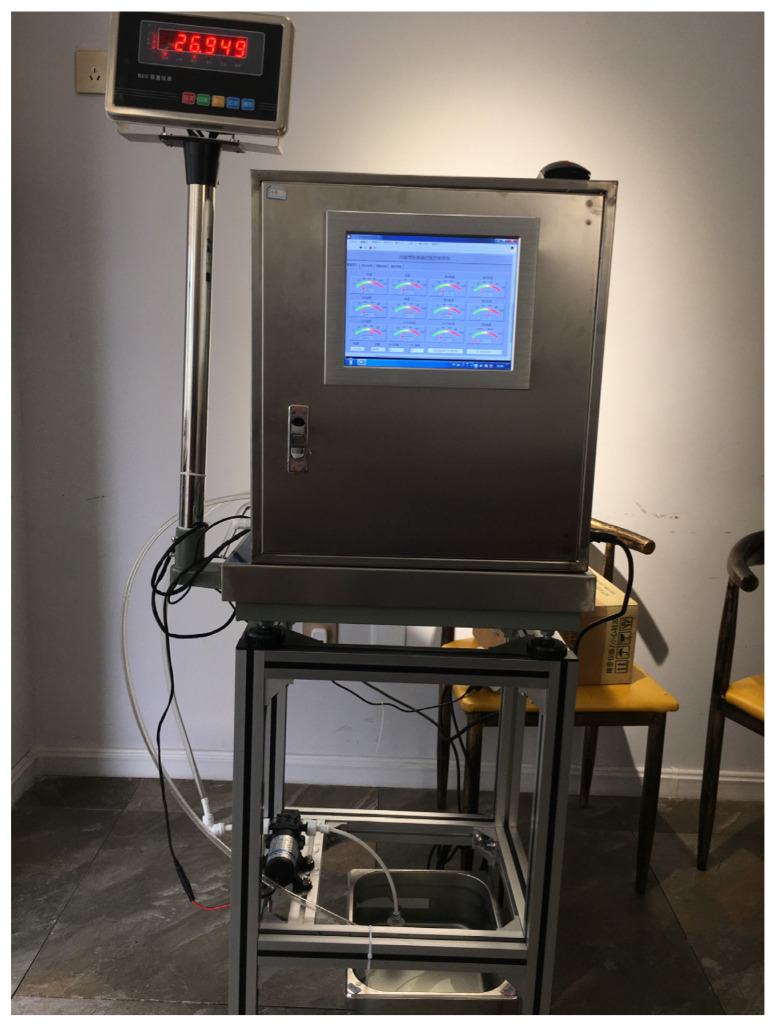
Fixed-bed drying test device of increased efficiency by condensation.

**Figure 2:**
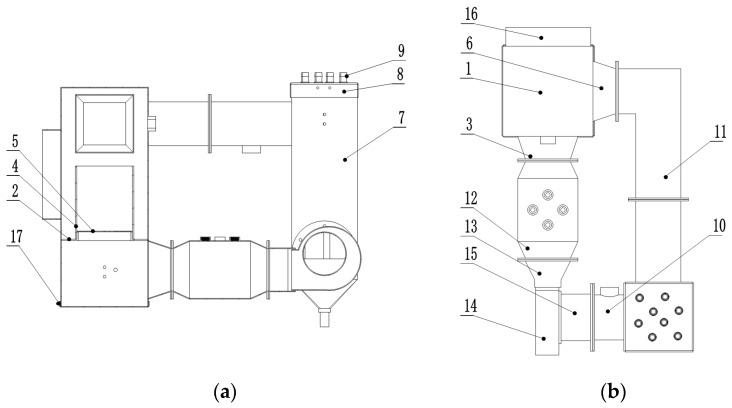
Internal structure diagram of test system. (**a**) Main section view and (**b**) top view. 1. Drying outer cylinder, 2. Drying inner cylinder support plate, 3. Drying outer cylinder lower duct, 4. Drying inner cylinder, 5. Drying inner cylinder bottom screen plate, 6. Upper duct, 7. Condenser, 8. Condenser upper cover, 9. Water cooler, 10. Condenser outlet duct, 11. Condenser inlet duct, 12. Heating duct, 13. Fan outlet duct, 14. Fan, 15. Fan inlet duct 16. Drying outer cylinder interface, 17. Drying outer cylinder bottom cover.

**Figure 3:**
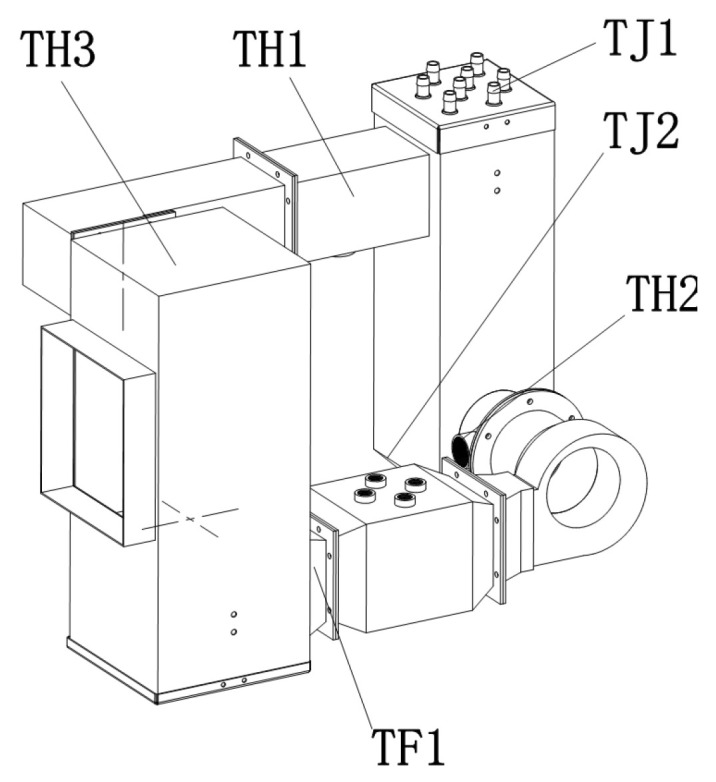
Sensor layout of test system. TF1: Hot air temperature; TJ1, TJ2: Condensing medium inlet and outlet temperature; TH1, TH2: Drying medium before and after condensation temperature and humidity; TH3: Drying chamber temperature and humidity.

The fixed-bed drying test device of increased efficiency by condensation was designed based on the principle of energy-saving drying of increased efficiency by condensation [25]. The drying medium is heated by the electric heater and sent to the drying chamber by the fan, where it exchanges moisture and heat with drying materials in the drying chamber; the drying medium that has absorbed the water from the grain enters the water cooling condensers for moderate condensation and dehumidification, and then enters the electric heater for supplementary heating to the required hot air temperature before proceeding to the next drying cycle. The drying medium flows in a closed loop inside the device. Figure 1 shows the fixed-bed drying test device of increased efficiency by condensation, which primarily consists of the test chamber, an electronic balance, a holder, and a water tank. Figure 2 shows the internal working components of the test device consisting of a drying chamber, a water-cooled condenser, an electric heater, a fan, detection sensors (Figure 3), and a control host, which are located inside the outer case. The inner wall of the outer case is insulated with 1 cm thick rubber plastic insulation cotton. The wet grains to be dried are dried in the drying chamber. The moisture in the grains evaporates and is exposed to the drying medium which is then discharged from the drying chamber in the form of humid exhaust gas. The humid exhaust gas is condensed and dehumidified when flowing through the condenser, and then flows in a closed loop in the system with the aid of the power provided by the fan. The heat source is an electric heater, which provides heat in a clean and eco-friendly manner. We can adjust the air velocity of the fan by controlling the main voltage, and the fan has a pulse speed measurement function. The detection sensors include the hot air temperature sensors, the water temperature sensors at the condenser inlet and outlet, and the temperature and humidity sensors before and after the condensation of the exhaust gas. The control host controls the “on” and “off” function of all system components, performs real-time monitoring, and stores the data. The control system, which was developed by LabVIEW, enables the window display of the control interface and the drying process parameter settings, as well as the display of the real-time interface. The models and parameters of the main components of the test device are shown in Table 1.

### 2.3. Test Principle

The energy consumption of the drying process, which is evaluated based on the specific heat consumption (SHC), is the main index to evaluate the energy saving of drying device and process. The SHC (measured in kJ/kg) is the amount of heat consumed per kilogram of water evaporated from the materials in the drying process [26]. Since the heat source of the test device is an electric heater, all the heat consumed for drying is provided by electricity, and the SHC computational equation is the following:

(1) $$SHC=\frac{3600W_{sr}}{W_{s}}$$

where $W_{sr}$ and $W_{s}$ are the electricity input consumed during electric heating (kWh) and the water loss (kg) observed during the drying process over the same time period, respectively.

The drying rate (μ), which is used to evaluate whether the drying is performed rapidly or slowly, is expressed by the variations in the moisture content (wet basis) of the materials per unit of time, and is calculated using the following equation [26]:

(2) $$\mu =\frac{M_{1}-M_{2}}{t}$$

where $M_{1}$ and $M_{2}$ are the moisture contents (%, wet basis) of the materials before and after drying, respectively, and t is the drying time (h).

Exergy is the amount of work a gas, liquid, or substance can perform when it is in a state that is not in equilibrium with a reference state [27], and is the unity of energy, environment, and sustainability. As the exergy efficiency increases in a process, the associated environmental effect decreases, whereas the sustainability increases [28]. The exergy efficiency in the drying process is the ratio of the exergy used to dry materials to the exergy of the drying medium in the inlet of drying chamber, and is calculated using the following equation [29]:

(3) $$\psi =\frac{{\dot{E}}_{\chi e}}{{\dot{E}}_{\chi a1}}$$

where ${\dot{E}}_{\chi e}$ is the exergy rate for the water evaporating from the grain, which was calculated as follows [29]:

(4) $${\dot{E}}_{\chi e}=\left(1-\frac{t_{0}}{t_{g}}\right){\dot{Q}}_{e}$$

where $t_{g}$ is the grain temperature and $t_{0}$ is the reference state temperature of the exergy analyses. The exergy reference state of the corn drying system in this study was considered as the condition of the drying chamber inside the case. ${\dot{Q}}_{e}$ is the heat required for the evaporation of grain water, which belongs to the useful energy of the system and was calculated as follows [30]:

(5) $${\dot{Q}}_{e}=n*\left(2500+1.842t_{a2}-C_{w}\theta_{g1}\right)$$

where *n* is a constant and it denotes water removal per unit time during the drying process, $t_{a2}$ is the exhaust gas temperature, $C_{w}$ is the specific heat by weight at a constant pressure of water, and $\theta_{g1}$ is the temperature of the corn before drying.

${\dot{E}}_{\chi a}$ is the exergy rate of the drying medium, and the subscript 1 and 2 refer to the state of the drying medium input to the drying chamber and exhaust gas discharged from it, respectively, which was calculated as follows [29]:

(6) $${\dot{E}}_{\chi a}={\dot{m}}_{a}\left\{\begin{array}{c} \left(C_{a}+\varphi C_{v}\right)\left(t-t_{0}\right)-t_{0}\left[\left(C_{a}+\varphi C_{v}\right)\ln\left(\frac{t}{t_{0}}\right)-\left(R_{a}+\varphi R_{v}\right)\ln\left(\frac{P}{P_{0}}\right)\right]\\ +t_{0}\left[\left(R_{a}+\varphi R_{v}\right)\ln\left(\frac{1+1.6078\varphi_{0}}{1+1.6078\varphi }\right)+1.6078\varphi R_{a}\ln\left(\frac{\varphi }{\varphi_{0}}\right)\right] \end{array}\right\}$$

where ${\dot{m}}_{a}$ is the mass flow rate of the drying medium during the experimental process; $C_{a}$ and $C_{v}$ are the specific heat of air and the mean specific heat of vapour, respectively; $R_{a}$ and $R_{v}$ are the gas constant and vapour gas constant equal to 0.287 kJ/kg·K and 0.462 kJ/kg·K, respectively; and t, P, and φ are the temperature, pressure, and humidity ratios of the drying medium, respectively.

The exergetic improvement potential rate and sustainability index are two useful parameters for analysing the exergy of a process. When the internal exergy loss in a system is reduced, the exergetic improvement potential is high; when the sustainability index in a process is high, the environmental effect is low [29]. The improvement potential rate and sustainability index are calculated using the following equations.

The improvement potential rate in the drying process is expressed as [29]:

(7) $$I\dot{P}=\left(1-\psi \right)\left(\dot{E}\chi_{in}-\dot{E}\chi_{out}\right)$$

(8) $$\dot{E}\chi_{in}=\dot{E}\chi_{g1}+\dot{E}\chi_{a1}$$

(9) $$\dot{E}\chi_{out}=\dot{E}\chi_{g2}+\dot{E}\chi_{a2}$$

where $\dot{E}\chi_{in}$ and $\dot{E}\chi_{out}$ are the exergy rate of the input and output drying chamber, respectively, and $\dot{E}\chi_{g}$ is the exergy rate of the grain, and the subscript 1 and 2 refer to the state of grain before and after drying, which was calculated as follows [29]:

(10) $${\dot{E}}_{\chi g}={\dot{m}}_{g}C_{g}\left[\left(t-t_{0}\right)-t_{0}\ln\frac{t}{t_{0}}\right]$$

where ${\dot{m}}_{g}$ and $C_{g}$ are the mass flow rate and the specific heat of the dried grain during the experimental process, respectively.

The sustainability index in the drying process is expressed as [29]:

(11) $$SI=\frac{1}{1-\psi }$$

### 2.4. Test Uncertainty Analysis

The reproducibility and validity of the data obtained during the corn drying test were verified by uncertainty analysis. The uncertainty or error of the temperature sensors and the temperature and humidity sensors mainly arises from the measurement accuracy of the sensor itself and the effect of the higher humidity where sensors were installed on its performance. The uncertainty of the load cell was affected by the air flow ambient environment. To check the accuracy of the thermocouple, grain moisture meter, and load cell, twenty repetitions of the test were performed. Temperature and humidity data of the exhaust air, corn temperature data, corn moisture data, and machine and grain weight data were collected over a time period. The mean value and standard deviation of all observed data were obtained. The variable X_i_ is uncertain and can be expressed as follows [31]:

(12) $$X_{i}=X_{mean}\pm \delta X_{i}$$

where $X_{i}$ is the actual value of the variable, $X_{mean}$ is the mean of the measurements, and $\delta X_{i}$ is the uncertainty in the measurement. The percentage of uncertainty is express as follows:

(13) $$\%\mathrm{Uncertainty}=\frac{\delta X_{i}}{X_{mean}}\times 100$$

The percent uncertainties of all instruments were calculated and are shown in Table 2. The percent uncertainty was in the range of 4.8%. According to Yamamura et al., for the reproducibility of an experiment, uncertainty values below 5% are considered acceptable [32].

## 3. Results and Discussion

### 3.1. Comparison between Drying of Increased efficiency by condensation and Conventional Hot Air Drying

**Figure 4:**
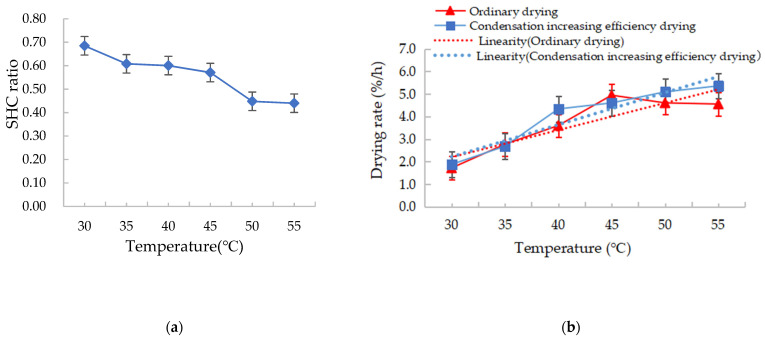
(**a**) Specific heat consumption (SHC) ratio and (**b**) drying rate of the two drying methods.

Hot air drying is commonly employed to dry grains, and hot air dryers occupy a market share of approximately 99%, having a thermal efficiency of approximately 70%. Plenty of heat is consumed during exhaust air emissions, and dehumidification plays a pivotal role in exhaust air recycling. Here, the water-cooled condenser of the device employed the waste heat of the exhaust air and the latent heat of condensation to condense and dehumidify the exhaust air discharged from the drying chamber for recycling, resulting in remarkable energy-saving effects. The drying characteristics during increased condensation efficiency drying and conventional hot air drying are compared in Figure 4.

When drying was performed at 30–55 °C, the SHC during the drying of increased efficiency by condensation was lower than that during a conventional open hot air drying process in the same temperature range. Specifically, the former was 0.68–0.44 of the latter, that is to say increased condensation efficiency drying resulted in an energy savings of 32–56% compared with the conventional open hot air drying; the energy-saving effect increased with increasing drying temperature. In Figure 4a, the SHC ratio refers to the ratio of the SHC between the drying of increased efficiency by condensation and conventional open hot air drying. Moreover, it can be seen from the trend line in Figure 4b that the mean drying rate during the drying process of increased efficiency by condensation was greater than that of conventional open hot air drying. This is mainly subject to the humidity and enthalpy of the drying medium, and the enthalpy of drying medium can be calculated by temperature and humidity parameters [30]. As the exhaust air is recovered during increased condensation efficiency drying, the moisture absorbed by the drying medium from the grains is not fully condensed in the condensation process. In other words, the moisture content and enthalpy of the drying medium during the drying of increased efficiency by condensation are higher than those during conventional open hot air drying at the same temperature. Therefore, although the drying medium of the drying process of increased efficiency by condensation has a high moisture content and is less capable of absorbing moisture than that of a conventional open hot air drying process under specific temperature conditions, it rapidly exchanges heat with the grains because of its high enthalpy, promoting moisture evaporation. This indicates that the high enthalpy of the drying medium is favourable to drying within a specific humidity range. It can be seen that exhaust air recycling can improve energy efficiency and reduce energy consumption, which is the same as the conclusion that exhaust air recycling can improve the exergy efficiency of the system concluded by Amantéa et al. [33]. It is also consistent with the research conclusions of Chen et al. who showed that corn dried at gradually increasing air temperature and humidity can obtain an optimal comprehensive drying goal, which includes energy savings and corn quality indices [34]. This is also the novelty of this experiment study.

### 3.2. Single Factor Experiment of Increased efficiency by condensation

#### 3.2.1. Effects of Hot Air Temperature on the Drying Characteristics

**Figure 5:**
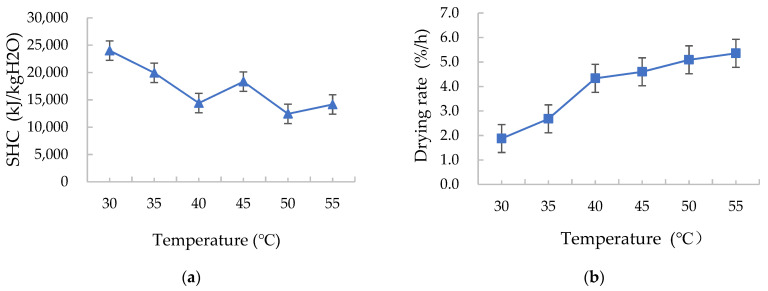
Effects of the hot air temperature on the (**a**) specific heat consumption (SHC) and (**b**) drying rate.

As the drying medium temperature in the inlet of drying chamber rose, the drying rate increased, whereas the SHC decreased, as shown in Figure 5. This is because when the air velocity, condensation ratio, and water temperature of the cooling medium are identical, the exhaust air temperature and dew point increase as the air temperature of the drying medium increases, and the condensation conditions can be reached, which means that condensation can occur earlier in the condensation process. This way, the relative humidity of the recovered exhaust air decreases earlier and remains at a low level. As a result, the drying medium is highly capable of absorbing moisture, resulting in a higher drying rate and a lower energy consumption. This is inconsistent with Ononogbo et al.’s research results from corn drying in a hot air dryer in the range of 50–60 °C, where the specific energy consumption increased with increasing air temperature [35]. This is also inconsistent with Zhang et al.’s simulation analyses of energy consumption in hot air drying of corn kernels in which energy consumption increased with increasing air temperature [36]. It is because the exhaust air recycling is used in this test device to make the drying system more energy-saving.

#### 3.2.2. Effects of the Air Velocity on the Drying Characteristics

**Figure 6:**
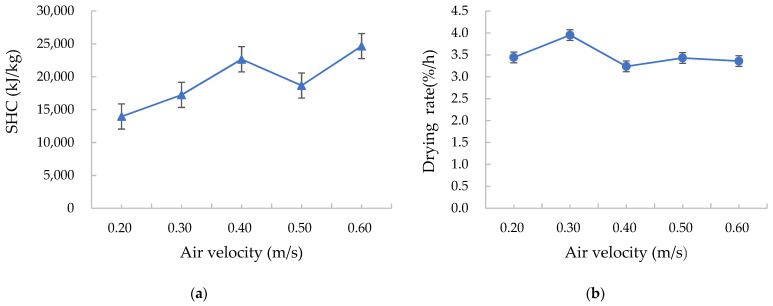
Effects of the air velocity on the (**a**) specific heat consumption (SHC) and (**b**) drying rate.

With the increase of the air velocity passed through the grain layer measured by the testo 512-1 differential pressure meter above the grain layer in the drying chamber, the SHC increased, whereas the drying rate first increased and then decreased and remained unchanged as the air velocity increased after a stable value was reached, as shown in Figure 6. This is due to the fact that the amount of air passing through the grain layer increased with an increasing air velocity; thus, the heat consumption required for reaching the pre-set air temperature was higher; the SHC of the drying process increased because the moisture absorbed from the grains did not change significantly. Furthermore, when the condensation ratio is constant, the cold fluid water flow, the amount of exchanged heat between the hot and cold fluids, and the heat loss increase with increasing air velocity. An air velocity value that is too high will cause heat waste; therefore, the air velocity value of the drying process should meet the drying needs. The influence of air velocity on specific heat consumption in the drying process is consistent with the research conclusions of Ononogbo et al. and Zhang et al. who showed that energy consumption increased with an increase in air velocity [35,36].

#### 3.2.3. Effects of the Condensation Ratio on the Drying Characteristics

**Figure 7:**
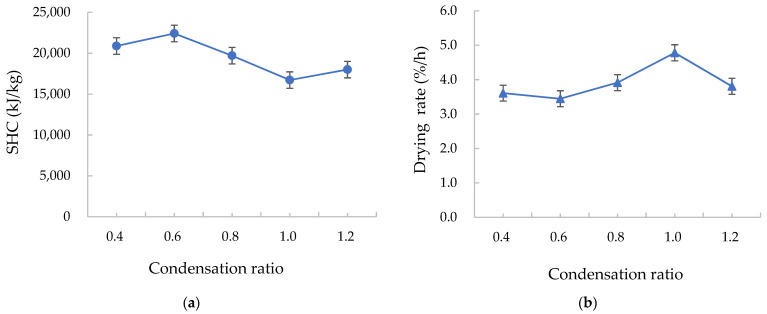
Effects of the condensation ratio on the (**a**) specific heat consumption (SHC) and (**b**) drying rate.

In the case of a low SHC and a high drying rate, an optimal condensation ratio was observed. Here, the condensation ratio is defined as the mass flow ratio between cold and hot fluids in the condenser. Under the test conditions, the SHC was the lowest and the drying rate was the highest when the condensation ratio was 1.0, as shown in Figure 7. Briefly, an optimal condensation ratio should be determined under specific operating conditions so that the exhaust air is condensed at an appropriate humidity to facilitate the drying process, and so that it is not excessively condensed to the point where it leads to an unnecessary energy waste. The process of controlling the humidity of the drying medium through the condensation ratio must meet the requirements of the drying index.

### 3.3. Rotation Combination Experiment Based on Response Surface Methodology

Grain drying is a non-linear, multi-coupled, and complex heat and mass transfer process, and the relationship between the drying process parameters and the drying characteristics is non-linear. Here, an orthogonal polynomial regression based on the SHC and drying rate values during the drying of increased efficiency by condensation was performed using response surface methodology, and the following regression equations were obtained:

(14) $$Y_{1}=3456.11994+126.66476X_{1}+30161.23338X_{2}-429.59375X_{1}X_{3}$$

(15) $$Y_{2}=1.49889+0.072053X_{1}+3.35118X_{2}-6.17892X_{3}-0.13750X_{1}X_{2}+0.13125X_{1}X_{3}$$

where *Y*_1_ and *Y*_2_ are the SHC and drying rate, respectively, and *X*_1_, *X*_2_, and *X*_3_ are the hot air temperature, air velocity, and condensation ratio, respectively.

ANOVA of the SHC and drying rate are shown in Table 3 and Table 4, respectively.

According to the above regression equations, the SHC in the drying process of increased efficiency by condensation is mainly dependent on the hot air temperature, air velocity, and the interaction between the hot air temperature and the condensation ratio, and the drying rate is primarily dependent on the hot air temperature, air velocity, condensation ratio, the interaction between the air temperature and air velocity, and the interaction between the air temperature and condensation ratio.

### 3.4. Study of the Exergy Characteristics of the Drying Process

#### 3.4.1. Effect of Drying Medium Temperature on the Mean Energy and Exergy Efficiencies of the Drying Process

**Figure 8:**
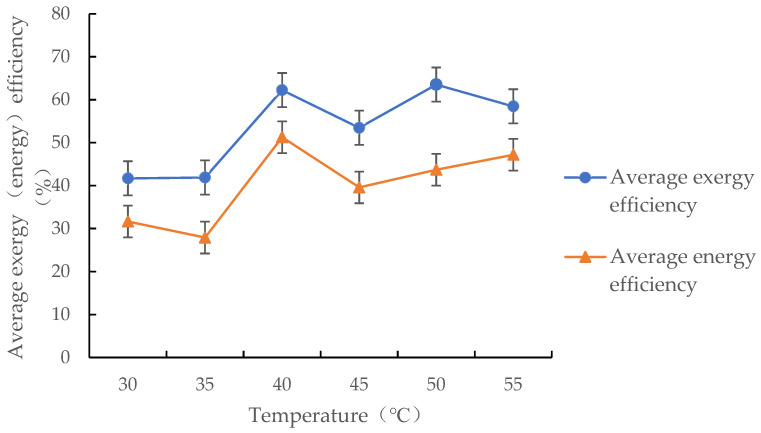
Changes in the mean energy (exergy) efficiency of the drying process with hot air temperature.

As shown in Figure 8, in the test temperature range, the mean energy and exergy efficiencies of the drying process were within the 31.65–51.26% and 41.69–63.52% ranges, respectively, and the mean exergy efficiency was greater than the mean energy efficiency. The mean energy and exergy efficiencies increased as the hot air temperature increased. This is mainly because the energy consumption required to evaporate 1 kg of water from the grains does not change significantly as the hot air temperature increases; however, the energy consumed in the drying chamber inlet increases as the hot air temperature increases. The change law of mean exergy efficiency with hot air temperature is consistent with the research conclusions of Khanali’s study on the plug flow fluidised bed drying process of rough rice and Mondal’s study on mixed flow drying of maize, in which exergy efficiency increased with an increase in drying air temperature [29,31]. However, our results are in contrast to Zohrabi’s study on convective drying of wood chips with exhaust air recirculation and Afzali’s study on infrared hot air drying of mushroom slices with air recycling system [5,6]. Thus, the change law of exergy efficiency with hot air temperature is related to drying mode.

#### 3.4.2. Effect of Air Velocity on the Mean Energy and Exergy Efficiencies of the Drying Process

**Figure 9:**
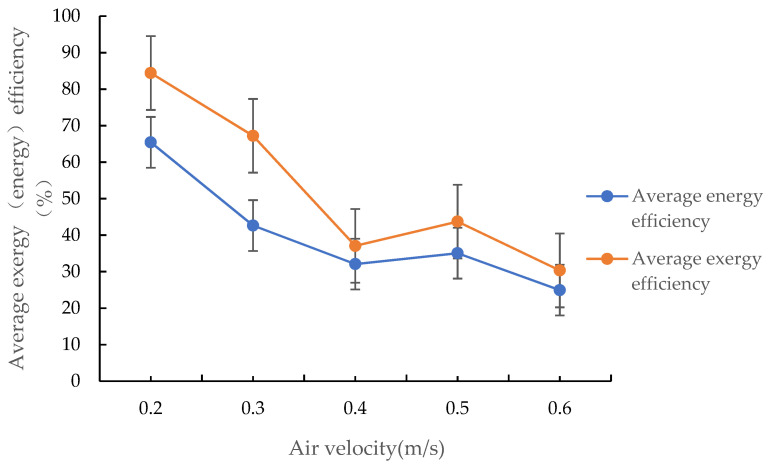
Changes in the mean energy (exergy) efficiency of the drying process with air velocity.

As shown in Figure 9, when air passes through the grain layer at 0.2–0.6 m/s, the mean energy and exergy efficiencies of the drying process of the increased efficiency by condensation of corn are in the 24.96–65.28% and 30.40–84.90% ranges, respectively, and both decreased with an increasing air velocity. This is primarily because the energy required to heat the drying medium increases with an increase in air velocity, since the thermal energy required to evaporate 1 kg of moisture from corn is essentially constant, the energy and exergy efficiencies of the drying process decrease with an increase in air velocity. Furthermore, an increase in air velocity leads to accelerated heat exchanges between the air duct and the inside of the box, between the inside and the wall of the box, and between the box and the external environment, leading to the dissipation of a higher amount of heat from the system and, as a result, the energy and exergy efficiencies decrease. This is the same as Tohidi et al.’s research results that energy efficiency increased with decreasing flow rate of the drying air [37].

#### 3.4.3. Effects of the Drying Medium Temperature on the Improvement Potential Rate and Sustainability Index of the Drying Process

**Figure 10:**
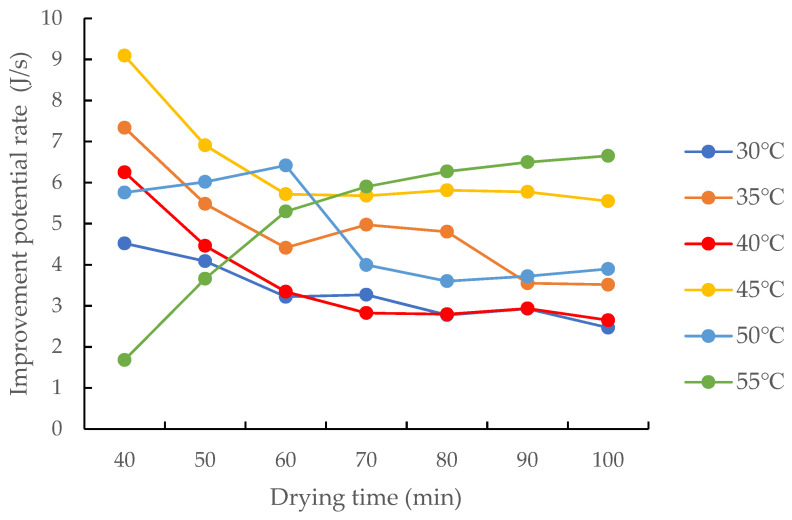
Changes in the improvement potential rate of the drying process at different hot air temperatures.

When the drying temperature was below 55 °C, the improvement potential rate decreased in the early drying process and, subsequently, tended to flatten out, as shown in Figure 10. This is mainly because the early drying process corresponds to the pre-heating stage of the drying system, and the exergy efficiency of the drying medium increases with an increasing in drying medium temperature. As the exergy efficiency of the grain drying system showed marginal changes with the changes in grain temperature, the exergy efficiency decreased in the early drying process. Moreover, since marginal changes in the differences between the initial and final grain temperatures and between the drying medium temperature and the exhaust air temperature occurred, the improvement potential rate of the drying process decreased. When the drying temperature was 55 °C, the improvement potential rate of the drying process gradually increased during the drying process. In the range of 30–55 °C, the improvement potential rate of the drying process had no obvious correlation with the change in drying air temperature. This is inconsistent with Khanali et al.’s study on the drying process of rough rice in a plug flow fluidised bed and Mondal’s study on mixed flow drying of maize, in which the improvement potential rate of the drying process increased with the increase in drying air temperature [29,31], which was mainly due to the effect of the condensation process on the drying process.

**Figure 11:**
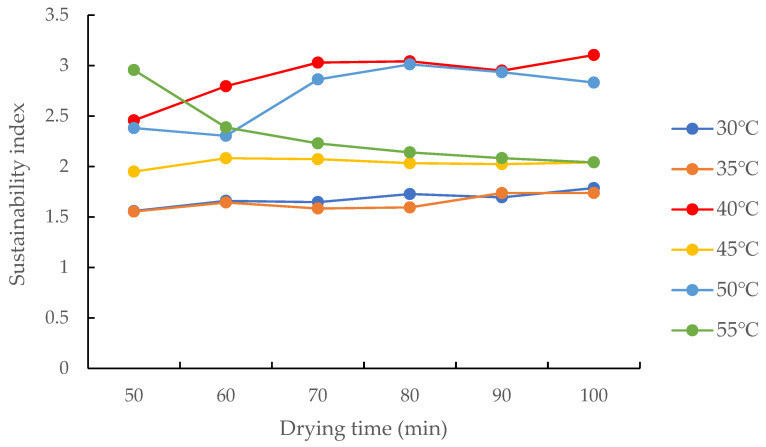
Changes in the sustainability index of the drying process at different hot air temperatures.

As shown in Figure 11, in the drying temperature range of 30–55 °C, the sustainability index of the drying process of increased efficiency by condensation fluctuated in the 1.56–3.10 range and showed no obvious pattern of change as the hot air temperature increased. This reveals that when drying is performed at 30–55 °C, the drying system does not exchange much energy with the external environment, and the heat dissipated by the system to the environment is maintained at a constant level and the sustainability index is marginally affected by the drying air temperature. This is inconsistent with Khanali et al.’s study about the drying process of rough rice in a plug flow fluidised bed and Mondal’s study on mixed-flow drying of maize, in which the sustainability index of the drying process increased with the increase in drying air temperature [29,31], which was mainly because the exhaust air of the test device is recycled and the drying medium flows in the closed loop of the system.

#### 3.4.4. Effects of the Drying Medium Flow Rate on the Improvement Potential Rate and Sustainability Index of the Drying Process

**Figure 12:**
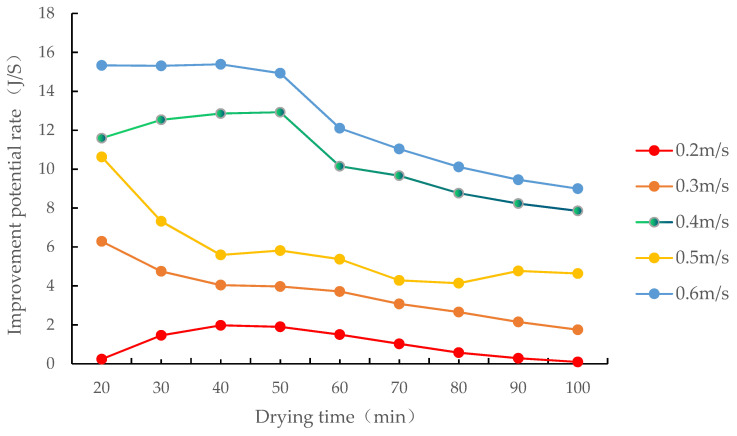
Changes in the improvement potential rate of the drying process at different air velocities.

The corn drying process and the condensation process of the drying medium influenced each other in the early drying process. As shown in Figure 12, as the drying process proceeded, the improvement potential rate showed no marked changes. When the drying was performed for nearly 1 h, the drying and condensation processes remained relatively stable, and the improvement potential rate of the drying process decreased as the drying process proceeded. In other words, the improvement potential rate determines when the system enters the equilibrium state of drying and condensation, representing the characteristic parameter of the system in the equilibrium state. When air passed through the grain layer at 0.2–0.6 m/s, the improvement potential rate of the fixed-bed drying process of increased efficiency by condensation of corn was in the 0.24–15.33 J/s range, and it increased as the air velocity increased. This is consistent with Beigi’s research on deep bed drying of rough rice in a convective dryer in which the improvement potential rate increased with the increase in flow rate [38].

**Figure 13:**
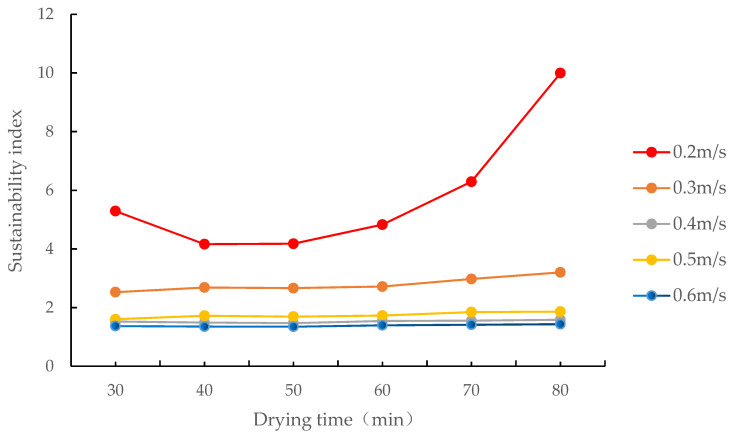
Changes in the sustainability index of the drying process at different air velocities.

When air passed through the grain layer at 0.4–0.6 m/s, the range of the sustainability index of the drying process was relatively narrow (1.3–1.9) and marginally fluctuated with an increase in drying time, as shown in Figure 13. When air passed through the grain layer at 0.3 m/s, the sustainability index of the drying process was within the 2.5–3.2 range. When air passed through the grain layer at 0.2 m/s, the sustainability index first decreased and then increased, and fluctuated in the 4.2–10.0 range during the drying process. Generally, as the air velocity increased, the sustainability index of the drying process decreased, and the effects of the drying process on the environment increased. This is consistent with Beigi’s research on deep bed drying of rough rice in a convective dryer in which the sustainability index decreased with the increase in flow rate [38].

## 4. Conclusions

In this study, we conducted a fixed-bed drying test of increased efficiency by condensation on corn to study the drying characteristics of this drying method and analyse the energy saving advantage and exergy characteristics of the drying process. The following conclusions were obtained:

(1) In the range of 30–55 °C, the SHC of corn drying using the drying of increased efficiency by condensation was 0.68–0.44 of that observed during conventional open hot air drying. Therefore, drying of increased efficiency by condensation resulted in an energy savings of 32–56% compared to conventional open hot air drying. Additionally, the energy-saving effects increased as the drying temperature increased, and the drying rate during increased efficiency drying by condensation was higher than that during conventional open hot air drying.
(2) When corn drying of increased efficiency by condensation was performed at 30–55 °C, the drying rate increased and the SHC decreased as the drying medium temperature increased. When air passed through the grain layer at 0.2–0.6 m/s, the SHC increased and the drying rate first increased, and then decreased before reaching a stable value as the air velocity increased.
(3) On corn drying of increased efficiency by condensation on a fixed-bed, when the drying air temperature was within the 30–55 °C range, the mean energy and exergy efficiencies were within the 31.65–51.26% and 41.69–63.52% ranges, respectively, and both increased with an increase in hot air temperature. When air passed through the grain layer at 0.2–0.6 m/s, they were within the 24.96–65.28% and 30.40–84.90% ranges, respectively, and both decreased with an increase in air velocity.
(4) The improvement potential rate and sustainability index of the drying process showed no obvious correlation with increasing drying air temperature, and the former increased and the latter decreased with increasing air velocity.

In future studies, the corn drying of increased efficiency by condensation should be performed at higher temperatures, and the drying characteristics and exergy characteristics should be analysed for the development of energy-saving drying processes, models, and control systems.

## 5. Patents

The measurement and control system V1.0 of the grain hot air drying and condensation enhancement experiment bench is based on the Labview platform; Chinese patent: 2022SR1534482.

Condensation heating grain drying basic test device, Chinese patent: 212030056U, 2019.

## Figures and Tables

**Table 1:** Models and parameters of the main components.

Name	Model	Parameters Specification
Control of the host	PPC-DL104D	10.4-inch industrial all-in-one machine
Aluminium water cooler	40 × 240 mm	Thickness 12 mm
Fan	BFB1012H	Air flow 0.712 m^3^/min, air pressure 249.082 Pa, motor speed 3600 rpm
Electric heating wire	24 v 25 W	Total power 100 W
Temperature sensor	PT100	Range −100–280 °C, precision 0.1 °C
Temperature and humidity sensor	HC2A-S	Humidity range 0~100% RH, temperature range −50~100 °C, precision ± 0.8% RH/0.01 °C
Power meter	DDSU666	0.001 kWh

**Table 2:** List of measuring instruments with their specification, accuracies, and uncertainties of the measured quantities.

Name of Instrument	Model	Accuracy	Standard Deviation	Uncertainty (%)
Temperature and humidity transmitter	HC2A-S	±0.005 °C ±0.8%RH	0.89 1.67	2.32 4.80
Temperature and humidity transmitter	TH10S-B-H	±0.2 °C ±2%RH	0.39 1.41	2.96 4.61
Temperature probe	PT100	±0.005	0.28	0.69
Electronic platform scale	—	±0.5 g	16.61	0.06
Grain moisture meter	PM-8188-A	±0.5%	0.18	1.31

**Table 3:** ANOVA of the model specific heat consumption.

Source	Sum of Squares (10^7^)	d f	Mean Square (10^7^)	F Value	*p* Value	Significance
Model	23.47	6	3.912	6.42	0.0025	Significant
X_1_	8.551	1	8.551	14.02	0.0025	
X_2_	10.37	1	10.37	17.01	0.0012	
X_3_	0.735	1	0.735	1.21	0.2922	
X_1_ X_2_	0.01412	1	0.01412	0.023	0.8814	
X_1_ X_3_	2.362	1	2.362	3.87	0.0707	
X_2_ X_3_	1.435	1	1.435	2.35	0.149	
Residual	7.927	13	0.6098			
Lack of Fit	4.063	8	0.5079	0.66	0.7156	Not significant
Pure Error	3.864	5	0.7727			
COR total	31.4	19				

**Table 4:** ANOVA of the model drying rate.

Source	Sum of Squares	d f	Mean Square	F Value	*p* Value	Significance
Model	20.95	6	3.49	26.71	<0.0001	Significant
X_1_	16.02	1	16.02	122.55	<0.0001	
X_2_	0.98	1	0.98	7.46	0.0171	
X_3_	0.97	1	0.97	7.39	0.0176	
X_1_ X_2_	0.60	1	0.60	4.63	0.0508	
X_1_ X_3_	2.21	1	2.21	16.87	0.0012	
X_2_ X_3_	0.18	1	0.18	1.38	0.2617	
Residual	1.70	13	0.13			
Lack of Fit	1.13	8	0.14	1.24	0.4229	Not significant
Pure Error	0.57	5	0.11			
COR total	22.65	19				

## Data Availability

The datasets generated for this study are available on request from the corresponding author.
